# Mammalian Target of Rapamycin Complex 2 Signaling Pathway Regulates Transient Receptor Potential Cation Channel 6 in Podocytes

**DOI:** 10.1371/journal.pone.0112972

**Published:** 2014-11-13

**Authors:** Fangrui Ding, Xiaoyan Zhang, Xuejuan Li, Yanqin Zhang, Baihong Li, Jie Ding

**Affiliations:** Department of Pediatrics, Peking University First Hospital, Beijing, China; Yong Loo Lin School of Medicine, National University of Singapore, Singapore

## Abstract

Transient receptor potential cation channel 6 (TRPC6) is a nonselective cation channel, and abnormal expression and gain of function of TRPC6 are involved in the pathogenesis of hereditary and nonhereditary forms of renal disease. Although the molecular mechanisms underlying these diseases remain poorly understood, recent investigations revealed that many signaling pathways are involved in regulating TRPC6. We aimed to examine the effect of the mammalian target of rapamycin (mTOR) complex (mTOR complex 1 [mTORC1] or mTOR complex 2 [mTORC2]) signaling pathways on TRPC6 in podocytes, which are highly terminally differentiated renal epithelial cells that are critically required for the maintenance of the glomerular filtration barrier. We applied both pharmacological inhibitors of mTOR and specific siRNAs against mTOR components to explore which mTOR signaling pathway is involved in the regulation of TRPC6 in podocytes. The podocytes were exposed to rapamycin, an inhibitor of mTORC1, and ku0063794, a dual inhibitor of mTORC1 and mTORC2. In addition, specific siRNA-mediated knockdown of the mTORC1 component raptor and the mTORC2 component rictor was employed. The TRPC6 mRNA and protein expression levels were examined via real-time quantitative PCR and Western blot, respectively. Additionally, fluorescence calcium imaging was performed to evaluate the function of TRPC6 in podocytes. Rapamycin displayed no effect on the TRPC6 mRNA or protein expression levels or TRPC6-dependent calcium influx in podocytes. However, ku0063794 down-regulated the TRPC6 mRNA and protein levels and suppressed TRPC6-dependent calcium influx in podocytes. Furthermore, knockdown of raptor did not affect TRPC6 expression or function, whereas rictor knockdown suppressed TRPC6 protein expression and TRPC6-dependent calcium influx in podocytes. These findings indicate that the mTORC2 signaling pathway regulates TRPC6 in podocytes but that the mTORC1 signaling pathway does not appear to exert an effect on TRPC6.

## Introduction

Transient receptor potential cation channel 6 (TRPC6) is a nonselective cation channel. In 2005, Winn *et al* and Reiser *at al* found that mutations in TRPC6 caused autosomal dominant focal and segmental glomerulosclerosis (FSGS) [1], [2]. In 2007, Moller *et al* found that both TRPC6 expression and function were increased in many acquired renal diseases, such as FSGS, minimal change disease (MCD) and membranous nephropathy (MN) [3]. These findings suggest the involvement of TRPC6 in the pathogenesis of hereditary and nonhereditary forms of renal disease. Therefore, maintaining TRPC6 expression and function at normal levels is a primary goal for the treatment of kidney diseases.

Although the exact mechanisms mediating TRPC6 regulation remain to be elucidated, more than one signaling pathway is involved in TRPC6 regulation. For example, angiotensin II contributes to podocyte injury by increasing TRPC6 expression via a nuclear factor of activated T-cells (NFAT)-mediated positive feedback signaling pathway [4], the NADPH oxidase-mediated ROS signaling pathway contributes to the up-regulation of TRPC6 expression in response to puromycin aminonucleoside-induced podocyte injury [5], and the Wnt/β-catenin signaling pathway mediates high glucose-induced cell injury via the of activation TRPC6 in podocytes [6]. In addition, in 2009, Beate *et al* found that rapamycin, which is an inhibitor of mammalian target of rapamycin (mTOR), regulates the expression of slit diaphragm proteins, including TRPC6, suggesting that the mTOR signaling pathway may affect TRPC6 [7]. However, due to differences in their complex components and substrates, mTOR complexes are categorized into mTOR complex 1 (mTORC1) and mTOR complex 2 (mTORC2) [8]. Thus, we sought to determine which mTOR signaling pathway regulates TRPC6 and the effect of mTOR signaling on TRPC6 function.

In our study, to further investigate the relationship between TRPC6 and mTOR signaling, we applied both pharmacological inhibitors and siRNAs specific for components of the two mTOR pathways to determine which mTOR complex signaling pathway is involved in regulating TRPC6 in podocytes. The results of this study may provide a new therapeutic target for renal diseases.

## Materials and Methods

### 1. Cell culture and drug treatment

The conditionally immortalized mouse podocyte cell line MPC5 used in our study was a kind gift from Professor Peter Mundel. MPC5 was firstly established in 1997 by Peter Mundel *et al*. Culture of MPC5 was performed as original described [9]. Briefly, the podocytes were cultured at 33°C in RPMI1640 medium (Gibco, USA) containing 10% fetal bovine serum (Gibco, USA), 100 U/ml penicillin-streptomycin (Gibco, USA) and 10 U/ml recombinant mouse interferon-γ (Sigma, USA). To induce podocyte differentiation, the cells were cultured at 37°C in the absence of interferon-γ for 10–14 days before experimental use. Rapamycin (Santa Cruz, USA) and ku0063794 (LC Laboratories, USA) were dissolved in dimethyl sulfoxide (DMSO) (Genechem, China), and the appropriate amount of DMSO was added to each control sample.

### 2. Real-time RT-PCR

Total RNA was extracted using TRIzol reagent (Invitrogen, USA) according to the manufacturer's instructions. The RNA concentration and purity were assessed using a NanoDrop 2000 Spectrophotometer (Thermo Scientific, USA). In total, 2 µg of RNA was reverse transcribed using the high-capacity cDNA Reverse Transcriptase (RT) kit (Invitrogen, USA) according to the manufacturer's protocol. The real-time RT-PCR mixture contained 2.0 µl of RT product, 10 µl of SYBR Green PCR mix (TransGen Biotech, China) and 0.5 µM primers in a final volume of 20 µl. The PCR mixture was denatured at 95°C for 5 min, followed by 40 cycles of melting at 95°C for 30 s, annealing at 64°C for 30 s and elongation at 72°C for 30 s. The primers used in real-time PCR were as follows:

mouse TRPC6 sense: 5′-TGGTGCGGAAGATGCTAGAA-3′;

mouse TRPC6 antisense: 5′-AAAGCATCCCCAACTCGAGA-3′;

mouse β-actin sense: 5′-AGCCATGTACGTAGCCATCC-3′; and

mouse β-actin antisense: 5′- GCTGTGGTGGTGAAGCTGTA-3′.

### 3. Western blot

Podocytes were lysed in lysis buffer (50 mM Tris-HCl (pH 7.4), 150 mM NaCl, 1% Nonidet P-40, 0.1% sodium dodecyl sulfate (SDS), and 0.5% deoxycholic acid sodium salt (DOC)). Equal amounts of total protein were separated on 10% gels via SDS-PAGE and electrophoretically transferred to nitrocellulose membranes (Amersham Life Science, USA). Nonspecific binding sites were blocked with 5% not-fat milk powder in PBS containing 0.05% Tween-20 for 1 h at room temperature. The membranes were incubated overnight at 4°C in one of the following primary antibodies at the appropriate concentrations: rabbit anti-TRPC6 (Alomone, Israel), rabbit anti-Akt (pan) (Cell Signaling Technology, USA), rabbit anti-phospho-Akt (Cell Signaling Technology, USA), mouse anti-phospho-p70 ribosomal S6 kinase (p70s6k) (Cell Signaling Technology, USA), rabbit anti-p70s6k (pan) (Cell Signaling Technology, USA), and mouse anti-β-actin (Santa Cruz, USA). Subsequently, the membranes were rinsed three times for 10 min each in PBS buffer containing 0.05% Tween-20 and incubated in horseradish peroxidase-conjugated anti-rabbit or anti-mouse IgG (Santa Cruz, USA). After a final washing step, the membranes were developed using an enhanced chemiluminescence reagent (Millipore, USA), and the specific protein bands were scanned and quantified relative to β-actin. Densitometric analysis of these images was performed using the ImageJ software (National Institute of Mental Health, USA).

### 4. siRNA-mediated knockdown of raptor and rictor

Specific siRNAs targeting mouse raptor and rictor (each siRNA product consists of a pool of 3 target-specific 19–25 nt siRNAs designed to knock down the gene expression) and non-targeted control siRNA were purchased from Santa Cruz (Santa Cruz, USA). Podocytes were seeded on a 6-well culture dish or coverslips on the preceding day to reach 50–60% confluence at the time of transfection. For each well, siRNA duplexes were diluted into 100 µl of serum-free medium. Then, 6 µl of lipofectamine 2000 (Invitrogen, USA) was added to 100 µl of the siRNA duplexes. The duplexes were incubated for 15 minutes and added to each well. After 48 hours of transfection, the cells were lysed for Western blotting or were used for calcium imaging.

### 5. Fluorescence calcium imaging

Podocytes were grown on coverslips and were then exposed to a drug (rapamycin, ku0063794, or DMSO) for 24 h or were transfected with siRNA. After removing the medium, the podocytes were washed three times with PBS and then incubated in 5 µmol/l Fluo-3 AM (DOJINDO, Japan) at 37°C for 20 min. After washing three times with PBS, the podocytes were incubated in PBS for 10 min to enable the calcium to completely bind to Fluo-3 AM. The fluorescence changes were detected via laser scanning confocal microscopy (Zeiss Lsm510 Meta, Germany). Fluo-3 AM fluorescence was excited at 480 nm and was detected at 510 nm. After the basal calcium fluorescence intensity was recorded, the cells on coverslips containing 0.5 ml of PBS were stimulated using 10 µmol/l hyperforin (Sigma-Aldrich, USA). Digital calcium images were captured at 3 s intervals. Three podocytes or fields were measured for three independent experiments, and the calcium fluorescence intensity values from the cells in each group were averaged.

### 6. Statistical analysis

When two groups of data were compared, the independent samples t-test was applied. All data and images were analyzed using the GraphPad Prism software, version 5 (San Diego, CA, USA). P-values less than or equal to 0.05 were considered to be significant, and all experiments were performed at least three times.

## Results

### 1. Rapamycin inhibits mTORC1, and Ku0063794 inhibits mTORC1 and mTORC2

**Figure 1:**
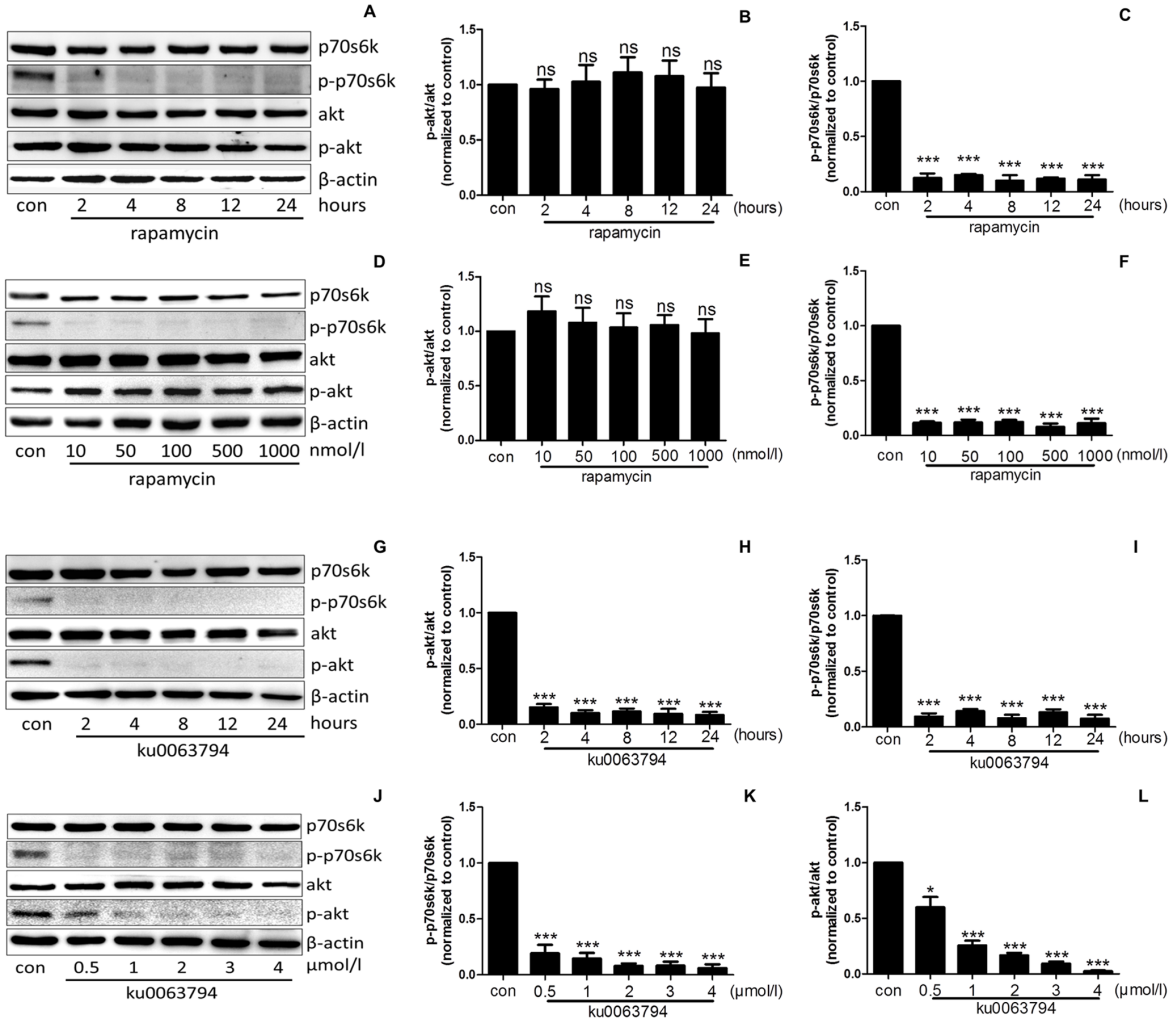
Effects of rapamycin and ku0063794 on downstream substrates of the mTORC1 and mTORC2 signaling pathways. (A–C) Rapamycin decreased the phosphorylation of the mTORC1 downstream target p70S6K in podocytes in a time-dependent manner. However, rapamycin did not decrease the phosphorylation of the mTORC2 downstream target Akt. (D–F) Rapamycin decreased the phosphorylation of p70s6k but not Akt in a concentration-dependent manner. (G–I) In a time-dependent manner, ku0063794, a dual inhibitor of mTORC1 and mTORC2, decreased the levels of the mTORC1 and mTORC2 downstream targets p-p70s6k and p-Akt, respectively. (J–L) In a concentration-dependent manner, ku0063794 at concentrations >1 µmol/l was sufficient to decrease both the p-p70s6k and p-Akt levels. (Abbreviations: con: control; p-p70s6k: phosphorylation of p70s6k; p-Akt: phosphorylation of Akt. *P<0.05 vs. control; **P<0.01 vs. control; ***P<0.001 vs. control; ns, no statistical significance; n = 3.)

mTORC1 activation results in the phosphorylation of its downstream target p70S6K, whereas mTORC2 activation results in the phosphorylation of Akt at Ser473 [10], [11]. Rapamycin not only affects mTORC1 signaling but also inhibits the assembly of mTORC2, such that prolonged rapamycin treatment suppresses the downstream activity of mTORC2 [12]. Based on a previous study, we chose a rapamycin concentration that does not exert this long-term effect to specifically examine the short-term effects of rapamycin [7]. Podocytes exposed to 50 nmol/l rapamycin for 2, 4, 8, 12 or 24 hours or to 10, 50, 100, 500 or 1000 nmol/l rapamycin for 24 hours were used to determine the rapamycin-induced decrease in the phospho-Akt (Ser473) (p-Akt) protein levels. No significant difference was detected in the p-Akt or Akt levels between each group (Figure 1A, B, D and E). However, the phospho-p70s6k (Thr389) (p-p70s6k) protein levels were markedly decreased in all groups (Figure 1A, B, C and F). No specific mTORC2 inhibitor exists; therefore, to evaluate the effects of mTORC2 signaling on TRPC6 expression, the podocytes were treated with ku0063794, a dual inhibitor of mTORC1 and mTORC2 [13]. As shown in Figure 1G–L, ku0063794 decreased the expression of both the downstream target of mTORC1, p-p70s6k, and the downstream target of mTORC2, p-Akt, in a time- and concentration-dependent manner.

### 2. Inhibition of the mTORC2 signaling pathway decreases the TRPC6 mRNA levels in podocytes

**Figure 2:**
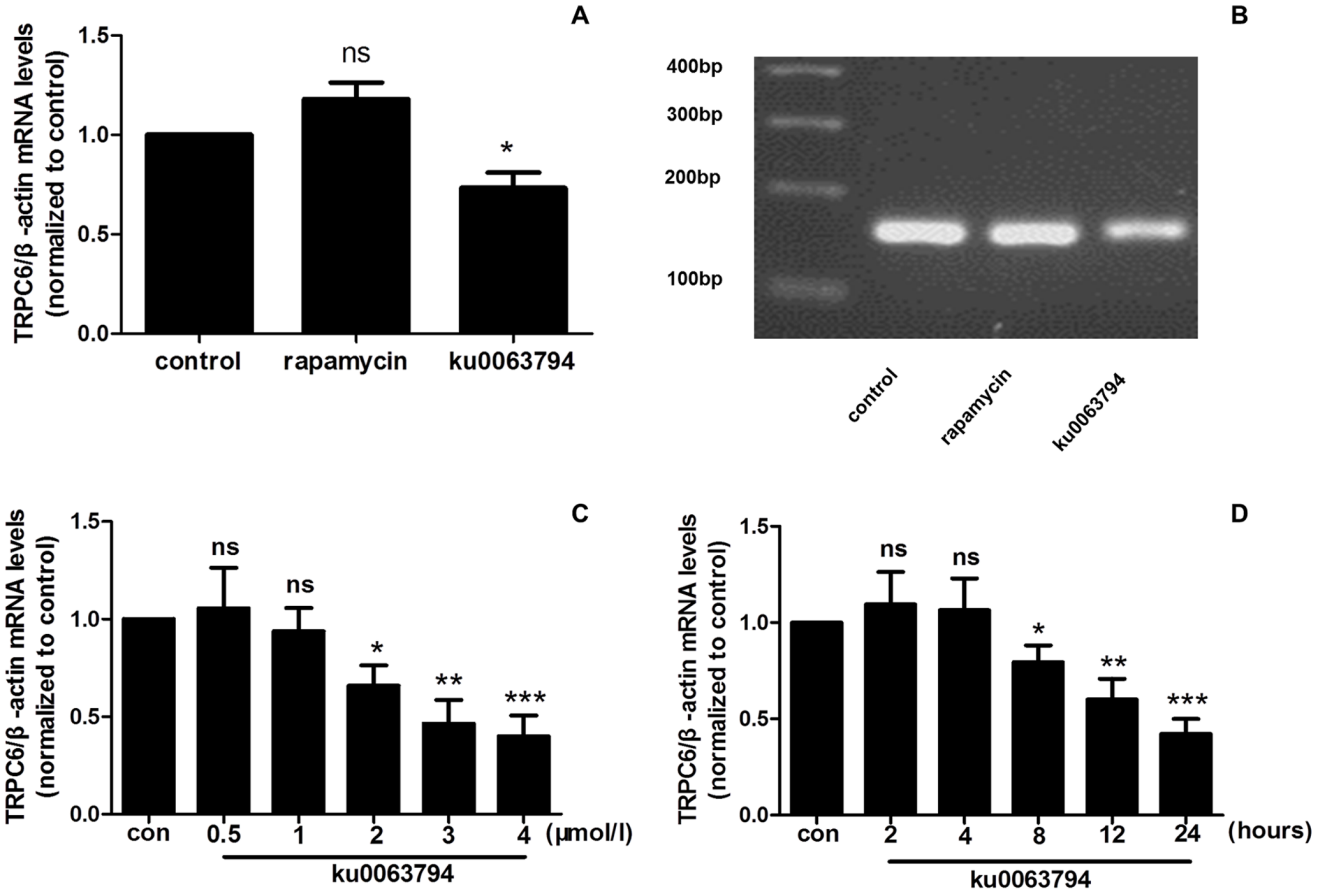
ku0063794 but not rapamycin decreased the TRPC6 mRNA levels in podocytes. (A) ku0063794 (2 µmol/l) or rapamycin (50 nmol/l) was applied to evaluate the effects of mTORC1 and mTORC2 on TRPC6 in podocytes. Application of ku0063794 for 24 h caused a significant decrease in the TRPC6 mRNA levels. However, no significant difference was detected between the control group and rapamycin treatment group. (B) The TRPC6 real-time PCR product was examined via agarose gel electrophoresis. Lane 1: 100 bp DNA marker; lane 2: control cells treated with DMSO for 24 hours; lanes 3 and 4: cells treated for 24 hours with 50 nmol/l rapamycin or 2 µmol/l ku0063794, respectively. (C) The TRPC6 mRNA levels were decreased in a concentration-dependent manner following treatment with 2, 3 or 4 µmol/l ku0063794. No significant difference between the control group and the 0.5 or 1 µmol/l ku0063794 treatment group was detected. (D) The TRPC6 mRNA levels were decreased following application of 3 µmol/l ku0063794 for 8, 12, or 24 h compared to the control treatment. There was no significant difference between the control treatment and ku0063794 treatment for 2 or 4 h. (*p<0.05 vs. control; **p<0.01 vs. control; ***p<0.001 vs. control; ns no statistical significance; n = 3.)

To determine which mTOR signaling pathway modulates the TRPC6 mRNA levels, podocytes were exposed to 50 nmol/l rapamycin or 2 µmol/l ku0063794. As shown in Figure 2A, rapamycin displayed no effect on the TRPC6 mRNA levels; however, ku0063794 caused a significant decrease in the TRPC6 mRNA levels. The specificity of the real-time RT-PCR amplification process for TRPC6 was confirmed by the appearance of a single band at 157 bp via gel electrophoresis (Figure 2B). Melting curve analysis confirmed the specificity of the TRPC6 transcripts in the podocytes. To further confirm this result, in podocytes exposed to 0.5, 1, 2, 3 or 4 µmol/l ku0063794 for 24 hours, the TRPC6 mRNA levels were decreased in a concentration-dependent manner (Figure 2C). In addition, the TRPC6 mRNA levels were decreased in a time-dependent manner when the podocytes were exposed to ku0063794 (3 µmol/l) for 2, 4, 8, 12 or 24 hours (Figure 2D).

### 3. Inhibiting the mTORC2 signaling pathway decreases TRPC6 protein expression in podocytes

**Figure 3:**
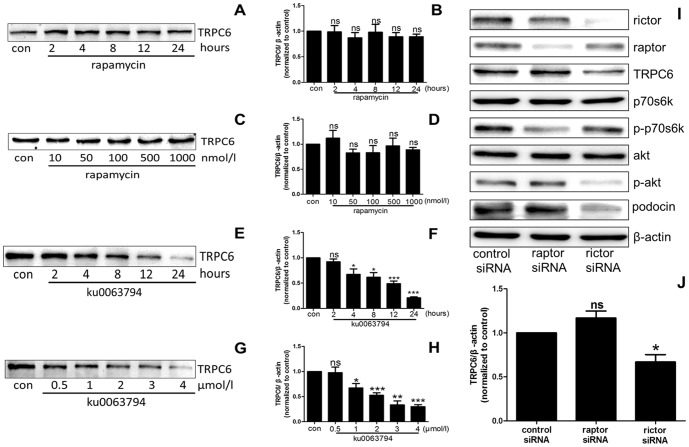
Inhibiting mTORC2 down-regulated TRPC6 protein expression. (A–D) In a time- and concentration-dependent manner, the TRPC6 protein levels displayed no significant difference between the control group and the rapamycin treatment group. (E, F) In podocytes exposed to ku0063794 for 2, 4, 8, 12, or 24 hours, the TRPC6 protein levels were decreased in a time-dependent manner. (G, H) The TRPC6 protein levels were decreased in a concentration-dependent manner following treatment with 0.5, 1, 2, 3, or 4 µmol/l ku0063794 compared to the control group. (I, J) The targeted siRNAs specifically knocked down raptor or rictor, accompanied by a decrease in the levels of the downstream target p-p70s6k or p-Akt, respectively. TRPC6 protein expression was decreased in the rictor siRNA treatment group compared to the control group. No difference was detected between the raptor siRNA and control siRNA treatment groups. Podocin protein expression was significantly decreased in rictor siRNA group while there was no difference in raptor siRNA group compared with control siRNA group. (*p<0.05 vs. control; **p<0.01 vs. control; ***p<0.001 vs. control; ns no statistical significance; n = 3.)

In podocytes exposed to 50 nmol/l rapamycin for 2, 4, 8, 12, or 24 hours or to 10, 50, 100, 500, or 1000 nmol/l rapamycin for 24 hours, no difference in TRPC6 protein expression was detected between the control and rapamycin treatment groups (Figure 3A–D). To evaluate the effect of mTORC2 signaling on TRPC6 expression, the podocytes were treated with ku0063794. As shown in Figure 3E–H, TRPC6 protein expression was significantly decreased in a time- and concentration-dependent manner. These data revealed that inhibition of the mTORC2 signaling pathway was involved in the decreased TRPC6 protein expression in podocytes. To completely clarify this result, we investigated TRPC6 protein expression using targeted siRNA knockdown. When siRNA specific for raptor, a component of the mTORC1 complex, was employed, TRPC6 protein expression was not different between the control siRNA and raptor siRNA groups. Transfection with siRNA specific for rictor, a component of mTORC2, caused a significant decrease in TRPC6 protein expression (Figure 3I–J). Based on these data, blocking the mTORC2 signaling pathway decreases TRPC6 protein expression in podocytes.

### 4. Inhibiting the mTORC2 signaling pathway decreases TRPC6-dependent calcium influx in podocytes

**Figure 4:**
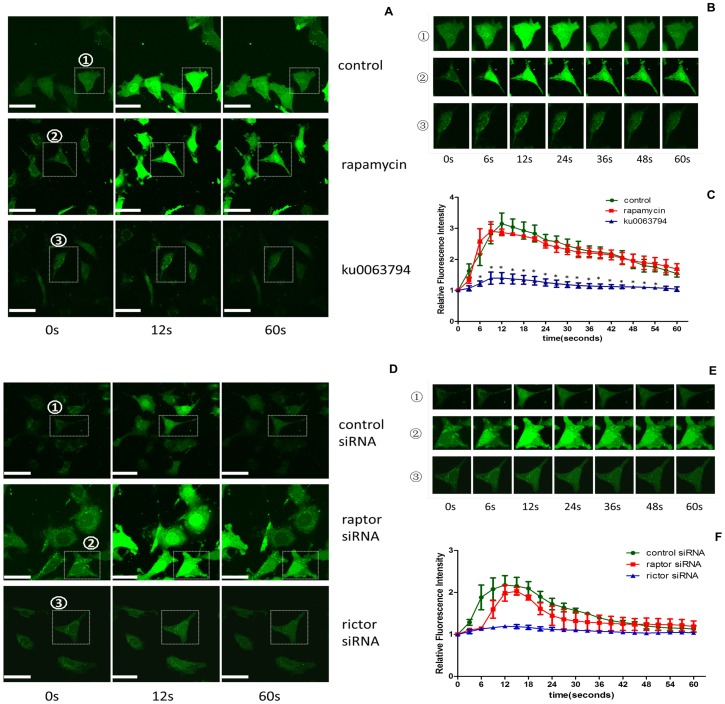
Blockade of mTORC2 decreased TRPC6-dependent calcium influx in podocytes (confocal microscopy). (A) The podocytes were imaged at 0 seconds before the addition of hyperforin, a specific agonist of TRPC6, to record the baseline fluorescence. The images in lines 2 and 3 were captured at the time point of the maximal fluorescence intensity (12-seconds) and at the last recorded time point (60-seconds) after hyperforin addition, respectively. The podocytes in rows 1, 2, and 3 were exposed to DMSO (control conditions), rapamycin (50 nmol/l) and ku0063794 (3 µmol/l) for 24 h, respectively. (B, E) The images in rows 1, 2, and 3 are magnifications of cells 1, 2, and 3 marked in Figure 4A, respectively, depicting the changes in fluorescence over shorter periods. Cell 1 and cell 2 exhibited similarly increased fluorescence within the first 12 seconds followed by decreased fluorescence beyond 12 seconds after hyperforin addition. Cell 3 exhibited no significant difference in fluorescence following hyperforin addition within 1 minute. (C, F) The calcium level in podocytes was expressed as the relative fluorescence intensity. Each trace represents the mean value that was derived from 3 cells or fields in a single experiment. The results are presented as the mean value of three independent experiments. (D) After treatment of podocytes with siRNA, the images were captured at 3 different time points: 0 s, 12 s and 60 s. The rictor siRNA group exhibited small changes in fluorescence intensity, whereas the control and raptor siRNA group exhibited peak-like changes in fluorescence intensity. (*P<0.05 vs. control; n = 3. Bar = 40 µm.)

TRPC6-mediated calcium influx plays an important role in renal diseases; therefore, the difference in TRPC6 function after treatment with pharmacological inhibitors or siRNA knockdown was also investigated in this study. The Fluo-3 AM fluorescence intensity, which indicates the calcium concentration, was evaluated in podocytes. To examine TRPC6-dependent calcium influx, the specific TRPC6 agonist hyperforin was applied in the present study [14]. As shown in Figure 4A–C, treatment with rapamycin (50 nmol/l) for 24 h did not change the hyperforin-induced Fluo-3 AM fluorescence intensity compared to the control. However, the hyperforin-induced Fluo-3 AM fluorescence intensity was significantly altered after treatment with ku0063794 (3 µmol/l) for 24 h compared to the control. Additionally, knockdown of raptor expression did not affect the Fluo-3 AM fluorescence intensity compared to control siRNA transfection, whereas of knockdown of rictor expression induced a significant alteration in the Fluo-3 AM fluorescence intensity compared to control siRNA transfection (Figure 4D–F).In addition, podocin protein expression was examined due to interact with TRPC6 and modulate the activation of TRPC6 [15]. As shown in Figure 3I, the expression of podocin was significantly decreased in the rictor siRNA group.

## Discussion

The mTOR protein, a serine/threonine kinase that belongs to the phosphoinositide 3-kinase (PI3K)-related kinase family, forms multi-protein complexes [16]. Based on the different complex components and substrates, the mTOR complex is categorized into mTORC1 and mTORC2 [8]. p70s6k is directly phosphorylated at Thr389 by mTORC1, and p-p70s6k is widely used to demonstrate mTORC1 activation in numerous studies [10], [17]–[19]. Alternatively, p-Akt is often used to indicate mTORC2 activation because Akt is directly phosphorylated by mTORC2 [11], [19]–[21]. In our study, the p-p70s6k and p-Akt levels were examined to assess mTORC1 and mTORC2 activity, respectively. As shown in Figure 1A, D, G, and J and Figure 3I, after treatment with inhibitors or siRNA, the p-p70s6k and p-Akt protein levels were significantly decreased, indicating the inhibition of the mTORC1 and mTORC2 signaling pathways, respectively.

In this study, we applied the pharmacological inhibitors rapamycin and ku0063794 to block the effects of mTORC1 and mTORC2 on TRPC6, respectively. Rapamycin was initially found to bind to the 12 kDa FK506-binding protein (FKBP12) and was subsequently found to inhibit mTORC1 function [22]. In recent years, many studies have reported that prolonged treatment with rapamycin also disassembles mTORC2, thereby inhibiting mTORC2 activity [12]. In our study, podocytes were exposed to various concentrations of rapamycin for different periods to examine the effects of this inhibitor on mTORC1 and mTORC2. The results revealed that rapamycin treatment decreased the p-p70s6k protein level; however, the p-Akt protein level remained unchanged after rapamycin treatment (Figure 1A and D). We conclude that rapamycin only inhibited the effect of mTORC1 on TRPC6 in our study. No specific inhibitor for mTORC2 currently exists; therefore, ku0063794, a dual inhibitor of mTORC1 and mTORC2 [13], was used to evaluate the effect of mTORC2 on TRPC6 in our study. If TRPC6 was only regulated by the mTORC1 pathway, the abnormal regulation of TRPC6 would be detected after treatment with rapamycin and ku0063794. Alternatively, if TRPC6 was only regulated by the mTORC2 signaling pathway, the abnormal regulation of TRPC6 would only be detected after treatment with ku0063794 but not rapamycin. According to the result shown in Figure 2A, we concluded that the inhibition of mTORC2 down-regulates the TRPC6 mRNA levels; however, mTORC1 does not affect TRPC6 expression. Additionally, as shown in Figure 3A–3H, inhibiting mTORC2 using ku0063794 decreases the TRPC6 protein levels.

In addition, our study utilized siRNAs specific for raptor and rictor to provide further direct effect on mTORC1 and mTORC2. mTORC1 includes mTOR, raptor, mLST8, PRAS40 and deptor, and mTORC2 includes mTOR, rictor, mSIN1, PRAS40, protor-1, and deptor [8]. In many studies, raptor knockdown induced an inhibitory effect on mTORC1 assembly and activity, whereas rictor knockdown induced an inhibitory effect on mTORC2 [23]–[28]. In our study, raptor and rictor were successfully knocked down using specific siRNAs for raptor and rictor (Figure 3I). Additionally, the p-p70s6k and p-Akt protein levels were decreased, indicating the inhibition of mTORC1 and mTORC2 after raptor and rictor knockdown, respectively. In addition, decreased TRPC6 protein expression was detected in the rictor siRNA group, directly indicating that inhibiting mTORC2 down-regulates TRPC6 protein expression; however, blocking mTORC1 signaling exerted little effect on TRPC6 protein expression (Figure 3I, 3J). Up-regulated TRPC6 protein expression occurs in many human glomerular diseases and in animal models of renal disease. TRPC6 was down-regulated at both the mRNA and protein levels after inhibition of the mTORC2 signaling pathway in our study, providing a potential therapeutic target for kidney diseases.

TRPC6 is a cation channel localized to the plasma membrane that plays a role in calcium entry into cells. In both hereditary and nonhereditary glomerular diseases, TRPC6 gain-of-function induces massive calcium influx into podocytes, and aberrant calcium levels disrupt the filtration barrier, promote the rearrangement of the highly dynamic podocyte actin cytoskeleton, and induce proteinuria [1], [3]. Our study also focused on the regulation of TRPC6 function. For this purpose, hyperforin was applied to evaluate specific TRPC6-mediated calcium influx, as previously reported [14]. As shown in Figure 4A–D, the reduced Fluo3 AM fluorescence intensity in the ku0063794 and rictor siRNA groups compared to the controls indicated decreased TRPC6 activation in podocytes, indicating that mTORC2 regulates TRPC6 function in podocytes. This finding suggests the potential of counteracting the gain-of-function effects of TRPC6 for the treatment of renal diseases. Surprisingly, the slight reduction of TRPC6 expression in response to ku0063794 and rictor siRNA caused dramatic changes on the calcium influx. According to Huber *et al* findings, podocin interacted with TRPC6 to regulate TRPC6 activity [15]. This finding made a potential contribution for explaining the dramatic calcium change caused by blockade of mTORC2. As shown in Figure 3I and Figure 4D, the inhibition of mTORC2 caused significantly decreasing of podocin along with the completely decreasing of the calcium influx. This result revealed that the decreasing of podocin induced by blockade of mTORC2 possibly related to the down regulation of TRPC6 function.

Interestingly, the podocyte morphology in the ku0063794 and rictor siRNA treatment groups was different from that of the other groups (Figure 4A, 4D). Although we did not quantify the morphological changes to the podocytes, the morphological differences between these groups were clear. This phenomenon may be induced by an mTORC2-mediated change in TRPC6 function. In our previous study, we reported that TRPC6 overexpression in podocytes induces cytoskeletal rearrangement by increasing intracellular calcium and activating RhoA [29]. A similar study reported that Gq protein-mediated activation of TRPC6 signaling induces RhoA activity and endothelial cell contraction [30]. mTORC2, a rapamycin-insensitive complex, was initially found to act upstream of Rho GTPases to regulate the actin cytoskeleton [31]. The cross-talk between mTORC2 and TRPC6 regulates the cytoskeleton via Rho GTPase. Although no direct evidence has elucidated the detailed mechanisms that regulate mTORC2 signaling and the actin cytoskeleton, according to the present results, the mTORC2 pathway may regulate the actin cytoskeleton via TRPC6-dependent calcium influx, which is essential for modulating the local activity of Rho GTPase. Further studies should focus on the effect of mTORC2 signaling on the actin cytoskeleton, as well as the mechanism underlying TRPC6-mediated calcium influx and Rho GTPase activity. In addition, because of the lacking of the kidney tissue from specific knockout mice for mTORC1 and mTORC2 and kidney biopsies from kidney transplantation patients treated with mTOR inhibitors, the conclusion in this study has not been confirmed by the *in vivo* data.

Although the role of mTORC2 signaling pathway in renal diseases was still under completely clarification, more and more studies revealed that mTORC2 was implicated in podocyte functions. Our study made a potential contribution to the molecular mechanism of mTORC2 signaling pathway involved in regulation the key ion channel TRPC6 in podocytes. In addition, the important role of mTORC2 in podocytes function was also reported. Gödel *et al* indicated that mTORC2 was essential for podocyte development and maintenance in diabetic nephropathy [32], and Canaud *et al* found that AKT2 activation by mTORC2 was required for podocyte survival in nephron reduction-induced renal disease [33].

In summary, our study demonstrated that inhibiting the mTORC2 signaling pathway down-regulates the mRNA and protein levels and function of TRPC6 in podocytes. We conclude that the mTORC2 signaling pathway is a novel regulator of TRPC6 in podocytes.
